# Different Processed Products of Curcumae Radix Regulate Pain-Related Substances in a Rat Model of Qi Stagnation and Blood Stasis

**DOI:** 10.3389/fphar.2020.00242

**Published:** 2020-03-20

**Authors:** Zhimin Chen, Lin Hu, Yujiao Liao, Xi Zhang, Zhuo Yang, Changjiang Hu, Lingying Yu

**Affiliations:** ^1^Chengdu University of Traditional Chinese Medicine, Chengdu, China; ^2^Chengdu Women’s and Children’s Central Hospital, Chengdu, China

**Keywords:** tuberous roots of *Curcuma longa* L. (i.e., Huangsiyujin, HSYJ), curcumins, β-endorphin (β-EP), 5-hydroxytryptamine (5-HT), c-fos

## Abstract

**Background:**

Curcumae blood Radix (Yujin) has been widely used to treat Qi stagnation and stasis in TCM. According to the Chinese Pharmacopoeia, the tuberous roots of *Curcuma longed* L. (i.e., Huangsiyujin, HSYJ) is one of the major species of Yujin. According to the processing theory of TCM, stir-frying HSYJ with vinegar might strengthen the effect of dispersing stagnated hepatoqi to relieve pain, and stir-frying HSYJ with wine might strengthen the effect of promoting blood circulation in order to remove blood stasis. However, the mechanism for the enhancement of clinical efficacy by processing is unclear.

**Aim/Hypothesis:**

This study was aimed at evaluating the effect of different processed products of HSYJ on chemical constituents and pain-related substances to explore underlying mechanisms of HSYJ in treating pain caused by Qi stagnation and blood stasis.

**Methods:**

The effects of different processing methods on the paste yield of water decoction were analyzed, and the content of the main constituents were detected by HPLC. A rat model of Qi stagnation and blood stasis was established by tail clamp stimulation combined with subcutaneous adrenaline injection. After treatment and intervention with HSYJ and its processed products, β-endorphin (β-EP) and 5-hydroxytryptamine (5-HT) were measured by ELISA, and the expression of c-fos was evaluated by immunohistochemistry.

**Results:**

After stir-frying with vinegar or wine, the extract yield and curcumin content increased. Compared with model group, raw HSYJ could significantly improve the abnormality of 5-HT in plasma (*P* < 0.05) and β-EP in brain (*P* < 0.01). Stir-frying HSYJ with vinegar or wine could significantly improve the abnormality of 5-HT in plasma, β-EP in brain, and the expression of c-fos (*P* < 0.01). Stir-frying HSYJ with vinegar could also significantly increase the level of β-EP in plasma (*P* < 0.05).

**Conclusion:**

These results showed that different processing methods have certain effects on the chemical constituents of HSYJ, mainly in increasing the decoction rate and curcumin content. HSYJ and its processed products can reduce 5-HT levels, increase β-EP levels, and inhibit the expression of c-fos in model rats. The effects of stir-frying HSYJ with vinegar on β-EP levels in plasma was superior to others.

## Introduction

Curcumae Radix (Yujin) is the dried root tuber of *Curcuma wenyujin* Y. H. Chen et C. Ling, *Curcuma longa* L., *Curcuma kwangsiensis* S. G. Lee et C. F. Liang, or *Curcuma phaeocaulis* Val. The drugs derived from the former two are known as “Wenyujin” and “Huangsiyujin,” respectively, and the drugs derived from the others are known as “Guiyujin” and “Lüsiyujin,” respectively, according to their different appearances. As one of the major species of Curcumae Radix, Huangsiyujin (HSYJ) is a well-known genuine traditional Chinese medicine in Sichuan. Its properties include activating blood, relieving pain, moving Qi, relieving depression, clearing the heart, cooling the blood, disinhibiting the gallbladder, and abating jaundice (Shiyuan, 2010; Chinese Pharmacopoeia Commission, 2015). It is widely used in Qi stagnation and blood stagnation syndrome (Tingmo, 2012). Modern pharmacological studies have shown that HSYJ has many functions, such as reducing inflammation (Jia et al., 2010; Chen-Xia et al., 2011), easing pain (Zhao et al., 2018), causing anti-thrombosis and anti-platelet aggregation (Jiang et al., 2015; Yongfeng, 2018), as well as being an antioxidant (Bengmark, 2006; Tuba and Ilhami, 2008), antidepressive, and cholagogue (Jiang et al., 2015).

The processing technology of Chinese herbal medicines is a traditional pharmaceutical technology based on the basic theory of TCM; this takes into consideration the differentiation of symptoms and the nature of drugs as well as the different requirements of dispensation and preparation. It is an important part of TCM, which has been accumulated and developed in clinical practice by TCM physicians. According to the processing theory of TCM, raw Yujin is good at relieving depression, by soothing the liver to regulate Qi, and relieving pain, by promoting blood circulation to remove blood stasis. Stir-frying with vinegar can concentrate the effects on liver meridian to strengthen the effect of dispersing stagnated hepatoqi to relieve pain; stir-frying with wine strengthens the effect of promoting blood circulation to remove blood stasis (Ye and Yuan, 2005; Administration, 2016).

In an earlier study, we found that HSYJ could increase the pain threshold, prolong latency of twisting, decrease writhing times, and influence hemorheology (Chen et al., 2019). Its processing mechanism, however, is not yet clear. This study was aimed at evaluating the effect of different processed products of HSYJ on chemical constituents and pain-related substances (β-EP, 5-HT, and c-fos protein expression) to explore the underlying processing mechanisms of HSYJ; the intention has been to provide reference for the further study, the development of Chinese patent medicine and clinical application of HSYJ.

## Materials and Methods

### Chemicals and Reagents

The reference standards of curcumin, demethoxycurcumin, and bisdemethoxycurcumin (purity > 98%) were purchased from the National Institutes for Food and Drug Control (Beijing, China). Methanol and acetonitrile (Fisher, United States) were of high-performance liquid chromatography (HPLC) grade. Other reagents were of analytical purity. Water was glass distilled and filtered through a Milli-Q water purification system (Millipore, Bedford, MA, United States) prior to use.

### Animals

SPF-grade Sprague-Dawley rats (200 ± 20 g) were obtained from Chengdu Dashuo Experimental Animal Co., Ltd. (Chengdu, China). Animals were housed in polypropylene cages and were sent to an animal room 5 days in advance for acclimatization (Chengdu University of Traditional Chinese Medicine, Chengdu, China). They were maintained under controlled conditions (a 12 h light-dark cycle at 22 ± 2°C) on a standard pellet diet and had free access to water. Animal experiments were approved by the Committee of Scientific Research and the Committee of Animal Care of Chengdu University of Traditional Chinese Medicine (Chengdu, China).

### Medicinal Materials and Reagents

Medicinal materials were collected from the genuine producing areas and authenticated as the dried root tuber of *Curcuma longa* L. by Professor LI Min (Chengdu University of Traditional Chinese Medicine, Chengdu, China). The sample information is shown in Table 1.

**TABLE 1:** Information of HSYJ samples.

No.	Production no	Place of Origin	Place of Purchase
H1	170701	Sichuan	Co.A
H2	17120804	Leshan (Sichuan)	Co.B
H3	XLS18030906	Qianwei (Sichuan)	Co.C

Xuefu Zhuyu Pian (Lot:170409) was purchased from Weifang Zhongshi Pharmaceutical Co., Ltd. (Shandong, China). Epinephrine Hydrochloride Injection (Lot:170507) was purchased from Grand Pharma (China) Co., Ltd. (Wuhan, China).

### Processing Methods of HSYJ

Prepared slices of HSYJ was processed according to the Pharmacopeia of the People’s Republic of China (Vision 2015) (Chinese Pharmacopoeia Commission, 2015) and the Standard for Processing of Traditional Chinese Medicine in Sichuan Province (Vision 2015) (Administration, 2016).

#### Raw HSYJ

Raw HSYJ was washed clean, moistened thoroughly, cut into slices, and dried.

#### Stir-Frying HSYJ With Vinegar

We took slices HSYJ, mixed them with a certain amount of rice vinegar evenly, moistened them thoroughly until the vinegar was absorbed completely, poured the drugs into a frying container, and fried them for 10 min (temperature 120–150°C) before we took them out and laid them down to cool. The amount used was 100 kg drugs with 15 kg vinegar. The vinegar was diluted with the same volume of water.

#### Stir-Frying HSYJ With Wine

We took slices HSYJ, mixed them with a certain amount of yellow wine evenly, moistened them thoroughly until the wine was absorbed completely, poured the drugs into a frying container, and fried them for 10 min (temperature 120–150°C) before we took them out and laid them down to cool. The amount used was 100 kg drugs with 10 kg wine. The wine was diluted with 1.5-fold water.

### Preparation of Decoction

The decoction was prepared according to the Standard for Management of TCM Decocting Room in Medical Institutions and Technical Requirements for Quality Control and Standard Formulation of TCM Granules.

HSYJ and its processed products were boiled for 30 min after soaking for 30 min with nine times the amount of water before being filtered. The dregs were boiled twice with seven times the amount of water for 30 min each time, and they were then filtered and mixed with the previous filtrate. The mixed decoction was concentrated by vacuum concentration (T ≤ 50°C). The dosage for rats was 0.9 g⋅kg^–1^ according to the conversion of human clinical dosage.

A positive drug was prepared by grinding up Xuefu Zhuyu Pian and dissolving this in water. The dosage given to the rat was 0.432 g⋅kg^–1^ according to the conversion of human clinical dosage.

### Extraction Yield

We took 50 mL of decoction and put it in an evaporating dish that had been dried to a constant weight. We then steamed it in a water bath, dried it in an oven at 105°C for 3 h, and cooled it in a dryer for 30 min. We then weighed it quickly and precisely and calculated the extraction yield. The extraction yield (%) of the sample was calculated with the dried product.

### Determination of Curcumins in Decoction

#### Sample Preparation

We took 50 mL of decoction and steamed it in a water bath (T ≤ 60°C), dissolved it in methanol of a fixed volume of 10 mL. This was then shaken and filtered with a filter (0.45 μm pore size) prior to injection.

#### Analysis Condition of HPLC

HPLC determinations were performed by using a SHIMADZU LC-2030C instrument (Shimadzu Corporation, Japan) equipped with a DAD detector, an auto sampler, a column heater, and a SHIMADZU Shim-pack GIST C18 (250 mm × 4.6 mm, 5 μm) column. The mobile phase was acetonitrile-4% glacial acetic acid aqueous solution (48:52); the detection wavelength was 425 nm; the flow rate was 1 mL⋅min^–1^; and the column temperature was 30°C.

### Trail Grouping and Animal Model Establishment

**FIGURE 1:**
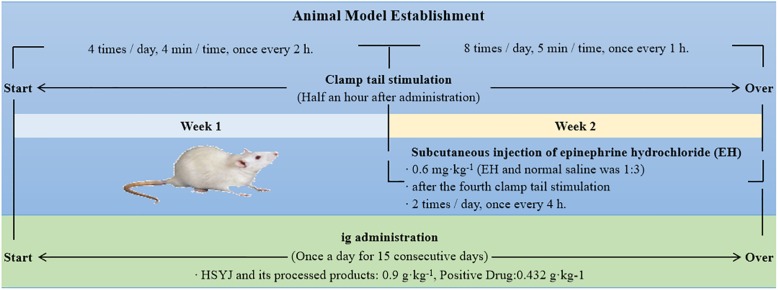
Diagrammatic sketch of the experimental process.

A total of 36 animals were randomly and equally divided into six groups (male and female in half): a control group (CG), model group (MG), Raw HSYJ group (SHG), Stir-frying HSYJ with vinegar group (CHG), Stir-frying HSYJ with wine group (JHG), and positive group (PoG). Except for the CG, the rest of the groups replicated the blood stasis model due to liver-Qi depression by the referenced methods (Shuzhi, 2014). When the model was finished, the animals were fasted for more than 12 h, but water was given normally. The samples were taken under anesthesia. The experimental procedure and dosing treatment plan are shown in Figure 1.

### Determination of 5-HT and β-EP by ELISA

We collected plasma using heparin as an anticoagulant and centrifuged it for 5 min at 3,000 rpm. The samples were stored in aliquot at −20°C for later use. We avoided repeated freeze-thaw cycles. Rat brain tissue samples were rinsed with normal saline and stored at −20°C. At the time of detection, rat liver tissue samples were thawed in 37°C water and weighed to the appropriate amount of tissue, ground with nine times the normal saline, and centrifuged for 10 min at 3,000 rpm. The supernatant was removed and assayed immediately. Rat 5-Hydroxytryptamine (5-HT) in plasma was detected with 5-HT ELISA Kit (Lot: C0167140192, Cusabio Biotech Co., Ltd., Wuhan, China). Rat beta-endorphin (β-EP) in plasma and brain were detected with β-EP ELISA Kit (Lot: C0167180196, Cusabio Biotech Co., Ltd., Wuhan, China).

### Immunohistochemistry Examination

The fixed brain was dehydrated by an automatic dehydrator before being embedded and sliced. The devoted slices were put in the dye vat and performed in 3% hydrogen peroxide (methanol and 30% H_2_O_2_ was 99:1) for 10 min at room temperature. Afterward, the slices were washed three times with PBS for 5 min each time. We then immersed them into 0.01M citrate buffer (PH 6.0), used high fire in a microwave oven to break off after boiling, and we repeated this one time after 5 min. After cooling, we washed them two times with PBS for 5 min each time. The slices were then incubated with normal goat serum (ZSGB-BIO, Beijing, China) at room temperature for 20 min. The sections were incubated with primary antibody for c-fos (rabbit polyclonal antibody, 1:100 dilution, Abcam, CA, United States) overnight at 4°C. The sections were then processed with the secondary antibody with goat anti-rabbit (ZSGB-BIO, Beijing, China) for 30 min at 37°C. We washed them three times with PBS for 5 min each time. Used DAB (ZSGB-BIO, Beijing, China) to develop color at room temperature, we controlled the reaction time under the microscope for 2 min and washed the sections with distilled water. Slices were slightly re-dyed with hematoxylin and sealed with neutral gum after dehydration and transparency.

Images were collected by a BA400Digital microscope (Motic China Group Co., Ltd.). The whole tissue was observed at 100 × magnification for each pathological section, and microscopic images were collected by selecting three fields of vision at 400 × magnification. Measured integrated optical density (IOD) and the area of all images by Image-Pro Plus 6.0 (Media Cybernetics, United States) and calculated mean density (MD) of each image.

### Statistical Analysis

Statistical analyses were performed by repeating one-way analysis of variance (ANOVA), and this was followed by LSD’s *post hoc* multiple-comparison test by using SPSS 19.0 software. The *P* < 0.05 was considered to represent statistical significance.

## Results

### Extraction Yield and Curcumins

**FIGURE 2:**
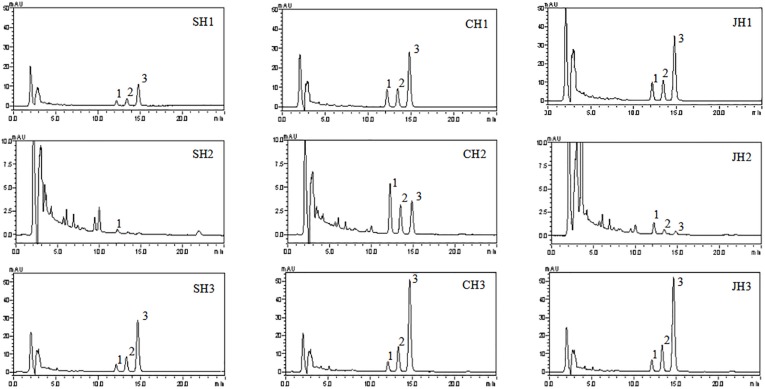
The chromatogram of HSYJ and its processed products 3.2 5-HT Level.

The extraction yield was significantly increased after being stir-fried with vinegar or wine, but there was no significant difference between the stir-frying HSYJ with vinegar and stir-frying HSYJ with wine, indicating that processing could increase the dissolution of chemical constituents (Table 2). Figure 2 shows the chromatogram of HPLC was well separated. After stir-frying with vinegar or wine, it was able to increase the dissolution of curcumins in water. The results are shown in Table 2. Similarly, there is no regularity in the content difference between stir-frying HSYJ with vinegar and stir-frying HSYJ with wine. The curcumins of H2 stir-frying HSYJ with vinegar were, however, much higher than those stir-frying with wine, which may be the source of the mechanism behind enhancing the analgesic effect of stir-frying with vinegar.

**TABLE 2:** Effects of different processing methods on the extraction yield and curcumins of HSYJ.

No.	Processing	Extraction Yield %	Bisdemethoxycurcumin (μg/g)	Demethoxycurcumin (μg/g)	Curcumin (μg/g)
H1	Raw HSYJ	13.83	1.237	2.460	7.344
	Stir-Frying With Vinegar	14.87	2.889	4.742	14.028
	Stir-Frying With Wine	14.43	3.048	5.293	15.689
H2	Raw HSYJ	9.99	0.162	–	–
	Stir-Frying With Vinegar	11.09	2.618	2.260	2.097
	Stir-Frying With Wine	11.64	0.403	0.334	0.104
H3	Raw HSYJ	9.02	2.386	8.594	30.128
	Stir-Frying With Vinegar	10.29	2.724	9.501	33.084
	Stir-Frying With Wine	10.12	3.051	9.889	33.907

**FIGURE 3:**
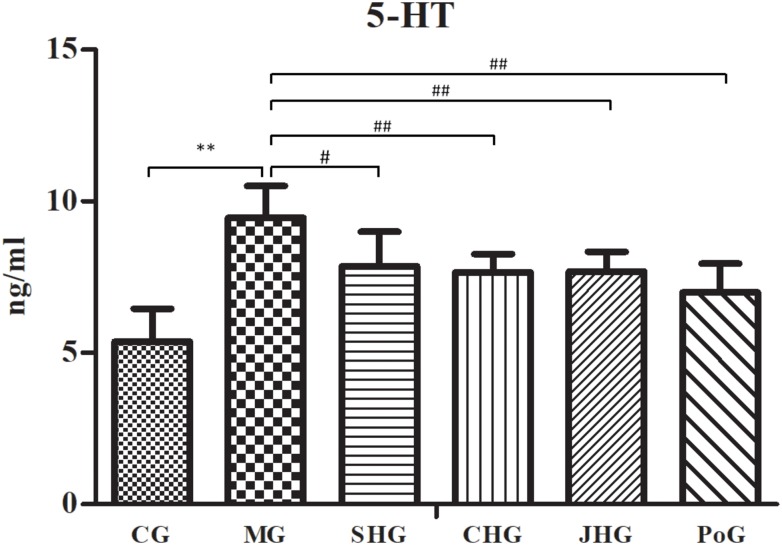
5-HT level in plasma analysis using ELISA (mean ± SD, *n* = 6). Results showed HSYJ and its processed products could significantly improve 5-HT abnormality in plasma of model rats. ^∗^*P* < 0.05 and ^∗∗^*P* < 0.01 vs. Control group; ^#^*P* < 0.05 and ^##^*P* < 0.01 vs. model group.

The results of changes to the 5-HT level are shown in Figure 3. Compared with the CG, the 5-HT level in plasma of MG was significantly increased (*P* < 0.01). After administration, HSYJ and its processed products could significantly improve 5-HT abnormality in plasma of model rats, but there was no difference between them.

### β-EP Level

**FIGURE 4:**
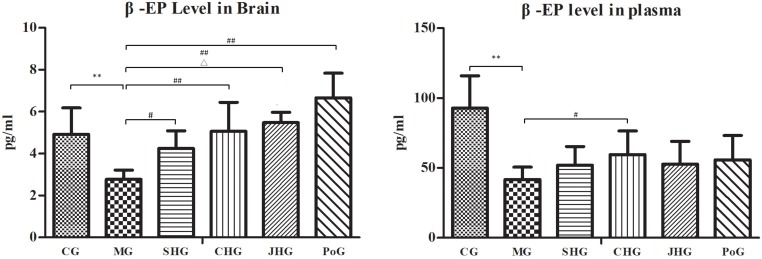
β-EP level in plasma and brain analysis using ELISA (mean ± SD, *n* = 6). Results showed HSYJ and its processed products could improve β-EP abnormality in plasma and brain of model rats. CHG has a significant effect on β-EP in brain and plasma. SHG and JHG only have significant effect on β-EP in plasma, but there is a significant difference between JHG and SHG. ^∗^*P* < 0.05 and ^∗∗^*P* < 0.01 vs. Control group; ^#^*P* < 0.05 and ^##^*P* < 0.01 vs. model group. ^△^ *P* < 0.05 vs. SHG.

The results of the changes to the β-EP level in plasma and brain are shown in Figure 4. Compared with the CG, the level of β-EP in plasma and brain of MG decreased significantly (*P* < 0.01). As an analgesic substance, the decrease of β-EP may be the mechanism behind Qi stagnation and blood stasis, causing pain. After administration, HSYJ and its processed products could improve β-EP abnormality in plasma and brain of model rats. Compared with MG, the β-EP in brain of HSYJ and its processed products increased significantly, and CHG and JHG were higher than those of GG. There was a significant difference between SHG and JHG. The results showed that HSYJ could enhance the analgesic effect by increasing the release of β-EP. The levels of β-EP in brain of drug groups were higher than that in CG, which may be due to the large release of β-EP in the brain in order to inhibit body pain. However, only the level of β-EP in plasma of CHG increased significantly, suggesting that stir-frying with vinegar not only increased the release of β-EP in brain but also promoted its entry into blood.

### C-fos Expression

**FIGURE 5:**
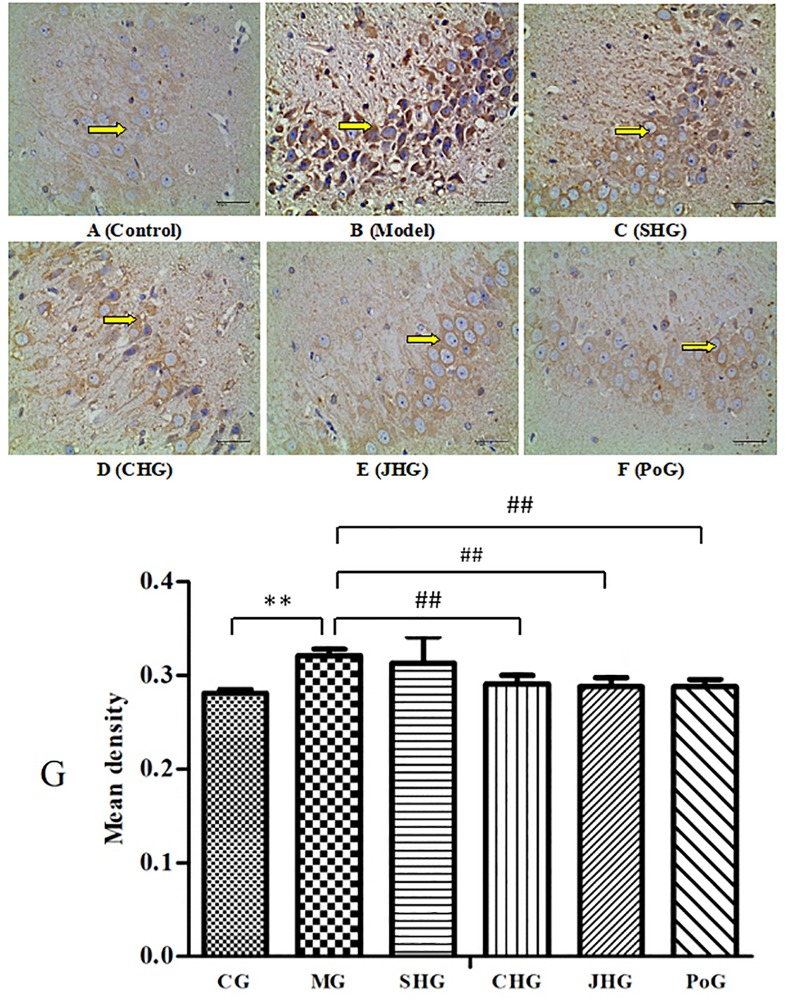
Expression of c-fos in rat brain analysis using immunohistochemistry (400×). **(A)** Control group, **(B)** Model group, **(C)** SHG group, **(D)** CHG group, **(E)** JHG group, **(F)** Positive group of c-fos immunohistochemistry staining. Bars **(G)** represent the means ± SD of 6 rats per group. Results showed CHG and JHG could improve c-fos abnormality. *P < 0.05 and **P < 0.01 vs. Control group; ^#^*P* < 0.05 and ^##^*P* < 0.01 vs. model group. ^△^*P* < 0.05 vs. SHG.

The results of the expression of c-fos in the brain of rats are shown in Figure 5. Compared with the CG, MG was significantly increased (*P* < 0.01). The indicator of the pain response is such that, the stronger the expression of c-fos, the stronger the pain the body experiences (Huang et al., 2010). After administration, the expression of c-fos in CHG and JHG decreased significantly (*P* < 0.01), but there was no significant difference in SHG, which indicated that HSYJ could enhance the analgesic effect of the body by stir-frying with vinegar or wine. There was, however, no significant difference in the administration groups.

## Discussion

As a unique pharmaceutical technology in China, the processing technology of Chinese herbal medicines plays an important role in improving the clinical efficacy of traditional Chinese medicine. Physical and chemical changes of Chinese Materia Medica will occur in the course of processing, and the pharmacological effects will be changed because of these changes, thus resulting in different clinical significance. Huangsiyujin, as a common medicine for Qi stagnation and blood stasis in TCM clinic, has a variety of processed products. Their functions are also dissimilar. However, few scholars have studied the differences of chemical constituents and pharmacodynamics of different processed products of HSYJ.

Traditional Chinese medicine clinically uses decoctions as medicines. In a broad sense, the extraction yield of decoctions can be used as one of the indexes to evaluate the quality of prepared slices of Chinese crude drugs. Modern research showed (Liu et al., 2018) that volatile oil and curcumins are the main chemical constituents of HSYJ. This has also been confirmed in our previous studies (Chen et al., 2019). In this study, the extraction yield and curcumins of HSYJ and its processed products were determined. The results showed that the HSYJ stir-frying with wine or vinegar was beneficial to the dissolution of chemical constituents such as bisdemethoxycurcumin, demethoxycurcumin, and curcumin in water decoctions. This may be because the texture of HSYJ becomes loose after stir-frying, which is more conducive to the dissolution of its chemical constituents. In addition, after stir-frying with wine or vinegar, it may promote the transformation of components and increase solubility.

The theory of TCM argues that stagnation of Qi may bring about pain. 5-HT is a strong pain-inducing substance (Wang et al., 2017, 2018). The elevated level of 5-HT in model rats is consistent with the TCM theory that “the pain is caused by obstruction.” β-EP, as one of the main bodies of endogenous opioid peptides, is produced by the hypothalamus and pituitary in vertebrates during exercise, excitement, pain, consumption of spicy food, and orgasm, and it resembles opiates in its ability to produce analgesia and a feeling of well-being (Chang-Hui and Shi-Jia, 2012; Zhang et al., 2016). Many studies have confirmed its analgesic effect, and it has become recognized as an inhibitory neurotransmitter to adjust pain (Qun et al., 2012; Du et al., 2016). The immediate early gene c-fos is frequently called the “third messenger” of the nucleus and is used to detect pathogenesis in central nervous system disorders. Under normal conditions, c-fos is present in the nucleus, but it is activated and transcribed to form mRNA in the cytoplasm and form fos proteins when the cells are subjected to various noxious stimuli (including pain, trauma, etc.) (Hongsheng and Liu, 2014). Now, a large number of studies have shown that the expression of c-fos can be interpreted as a sign of the excitement of painful neurons, and it is thus a useful tool for pain research (Huang et al., 2010; Hongsheng and Liu, 2014). Therefore, detection of β-EP and c-fos can determine whether there is pain neuron excitation in the rat body and can assess the body’s feedback. The results show that the analgesic mechanism of HSYJ may be to regulate the release of 5-HT, increase the content of β-EP, especially the release of β-EP in brain tissue, and inhibit the transmission of nociceptive pain information by inhibiting the expression of c-fos proteins in order to finally achieve the analgesic effect. After stir-frying with vinegar or wine, the effect becomes enhanced. This may be due to the fact that stir-frying with vinegar might help to concentrate the effects on liver meridian to strengthen analgesic effect by dispersing stagnated hepatoqi, and stir-frying with wine might strengthen the effect of promoting blood circulation for removing blood stasis; the body’s meridians become normal, and so the body does not feel pain or discomfort. This study provides reference for the further study, the development of Chinese patent medicine and clinical application of HSYJ. We will also continue to conduct in-depth research on its mechanism from other aspects.

## Conclusion

This study indicated that different processing methods have certain effects on the chemical constituents of HSYJ, and this is achieved mainly through increasing the extraction yield and content of bisdemethoxycurcumin, demethoxycurcumin, and curcumin. Modern research showed that curcumin might play an analgesic role through a variety of mechanisms and alleviate different types of pain. HSYJ and its processed products may alleviate pain by regulating the release of 5-HT, increasing the content of β-EP and inhibiting the expression of c-fos. Stir-frying HSYJ with vinegar is more conducive to the release and blood entry of β-EP, thus enhancing the analgesic effect of the body, which is consistent with the traditional Chinese medicine theory of “stir-frying with vinegar into liver to strengthen the effect of dispersing stagnated hepatoqi to relieve pain.”

## Data Availability Statement

The datasets generated for this study are available on request to the corresponding author.

## Ethics Statement

This study was conducted in strict accordance with the recommendations of the Guidelines for the Care and Use of Laboratory Animals of the Ministry of Science and Technology of China. The protocol was approved by the Committee of Animal Care of Chengdu University of Traditional Chinese Medicine (Chengdu, China).

## Author Contributions

ZC overall responsibility for the manuscript. LH revised the manuscript and polished the language. YL, XZ, ZY, and LY performed the experiments. CH was involved in the design of the research.

## Conflict of Interest

The authors declare that the research was conducted in the absence of any commercial or financial relationships that could be construed as a potential conflict of interest.
